# The framework and features of language policies in global constitutional texts

**DOI:** 10.3389/fpsyg.2022.1064034

**Published:** 2023-01-04

**Authors:** Chen Zhang, Ronghui Zhao, Yan Huang

**Affiliations:** ^1^China Center for Language Planning and Policy Studies, Shanghai International Studies University, Shanghai, China; ^2^School of Foreign Languages, East China University of Science and Technology, Shanghai, China

**Keywords:** language policy, framework, feature, constitutional texts, combined qualitative/quantitative method

## Abstract

Language policy, which is directly concerned with language practice, language ideology and language management, has become increasingly important in real social life. Explicit language policies in different fields, such as texts in law, education, and the public, have been explored for many years. However, the global comparative research on language policies in various constitutional texts (CT) is quite limited. In response, the present study aimed to investigate the framework and features of language policies in global CT through a combination of qualitative and quantitative analysis. Results showed that: 1) there were seven parts of the CT dealing with language policies, such as *Preamble*, *General principle*, *The state*, *Fundamental rights* and *Duties of citizen*, *State authority*, *National objectives*, and *Supplementary*; 2) there existed significant differences in the frequency of language policies in seven parts of the CT. Among them, language policies appeared most frequently in the part of *Fundamental rights and duties of citizen*; 3) the geographical location where the Constitution was enacted affected the distribution of language policies across parts. Overall, our findings suggested that the language policy in CT was influenced not only by constitutional principles, but also by the national language environment.

## Introduction

Generally, policies are framed in various fields by the lawmakers to provide the executive with a roadmap. Language policy is considered to be one of the most important among the many policies formulated in various fields of life such as economy, health, education, environment, social security, industry and trade (Kumar, 2020). Therefore, policy can be viewed as an ensemble of activities, some of which are textual (laws, reports, authorisations; Lo Bianco, 2008). Walsh (2012) enriched and deepened the understanding of the theoretical framework of language policy through the analysis of texts in language schemes. The Constitution, which provided a legal framework for the country to formulate policies in various fields such as legislative, executive, and judiciary fields, was the most powerful texts for its compliance with national ideology and guidelines. In fact, language policy and the Constitution are entwined, wherein the latter acts as guiding entity. Language policy usually refers to the rules or laws that determine the usage, status, and rights of a language(s) in a country. Spolsky (2004) once pointed out the main features of the theory of language policy. The first point was the tripartite division of language policy into language practices, language beliefs and language ideology. The second feature was that language policy functioned in complex relationships. Constitutional texts (CT), as the expression form of language policy, were also permeated with ideas, ideals, and ideologies (Frankenberg, 2006). CT would be a good example of language policy and the study of it would reflect basic features of Language policy.

Understanding the language ideology patterns in the texts of national constitutional language policy could help us to clarify the relationship between language ideology, language management and other theoretical structures of language policy. For example, language ideology pattern could reveal whether language-ideological changes in language management are systematic. In addition, it could examine the similarities and variability in the constitutional sources of ideological production in countries around the world. In short, it helps us to enhance our understanding of the internal functions of texts and the external ideology of language policy. Furthermore, such research reveals whether legal text types are better at conveying ideological information to policy agents than other text types in terms of state ideology. This is important since policy-makers access texts more frequently than others and use different texts for different goals. Thus, trans-constitutional language ideologies might influence the overall impact of legal frameworks on language policy.

Meanwhile, an increasing number of researchers have studied the language policies of the Constitution (Verschik, 2005; Garibova and Asgarova, 2009; Ó Flatharta, 2015; Sokolova et al., 2019). Indeed, the language policies of the Constitution that directly or indirectly deal with national symbols, solidarity, linguistic equality and protection are important for understanding the norms and principles of the Constitution. Additionally, policies on how to regulate relationships between different groups, especially ethnic minorities (Ruiz Vieytez, 2004; Kadenge and Kufakunesu, 2018), usually reflected in the language regulations. As mentioned earlier, the understanding of texts at the national level is more complex and inextricably linked to identity and power, reflecting the linguistic institutions and traditions of each country. For instance, Malan (2008) has suggested that the South African Parliament should not violate the language provisions of the Constitution in the legislative process, otherwise it would damage the crucial cultural assets of the South African citizenry. In summary, we found that the study of language policies was no longer confined to the constitutional provisions itself but was closely related to the access of citizens to human rights and the safeguarding of state power.

Based on Lo Bianco’s (2008) concept of language policy texts in the constitutional domain, our study aimed to find a framework of language policies in CT and to analyze the language features of the Constitution across different parts in form of CT and countries, which could provide a new research perspective for the Language Policy and Planning (hereinafter LPP) theory.

## Literature review

### Language policies in CT from linguistic and legal perspectives

The analysis of language policies in the Constitution is often politically and legally relevant. Powell (2018) pointed out that “Some discussions of language rights refer to constitutional provisions (Asmah, 1971) or international human rights covenants (Phillipson, 1992); others (Cooper, 1989; Tollefson, 2002) go a step further and argue that it exploits language conflict for political purposes. It is naive to expect the legal system, as a state-established authority, to be isolated from politics.” To a large extent, language policy making exists in the issuance of laws and in the legal-political practice of regulation. LPPs in this sense constitute the body of declarations proclaimed by authoritative bodies (Lo Bianco, 2009). Constitutions are thus the most overt and declared mode of language planning, the ultimate public text, formal operations involving laws, regulations, and planning and implementation. However, from the perspective of LPP, language use is rarely used as an administrative activity or the object of language analysis. Such public texts in countries around the world have not been analyzed as a whole language practice. Fitzsimmons-Doolan (2019) expanded the legal text into a special register, resulting in a deeper understanding of the field of language policy. Therefore, on this basis, we can study the language practice of large-scale CT in the legal field.

Linguistic and legal perspectives are often the main theoretical perspectives for studying CT, and researchers in both fields have made significant contributions. Previous studies have described contents dealing with language policies in the Constitution, such as the gap between language practices and language provisions (Gafaranga et al., 2013), the role of language institutions (Rousseau and Dargent, 2019) in accordance with legal principles, especially more emphasis has been placed on the dominant role of language power in state institutions (Meeuwis, 2015), the influential factor of constitutional bilingualism (Saarinen, 2018) on civic attitudes and national identity (Korhecz, 2002) and the constitutional effect on nation building (Charles, 2015).

In addition, some researchers have also paid attention to the comparative analysis of the Constitutions across countries. For example, Ruiz Vieytez (2004) conducted a jurisprudential analysis of the terms “official language” and “national language” in the Constitutions of 48 European countries. Lagarde (2019) compared the legal concept of minorities in terms of linguistic pluralism of French and Spanish CT. Besides, the contradiction between the implementation of language policy and the language norms in the Constitution (Kużelewska, 2015; Jiménez-Salcedo, 2019) also has practical significance. On this basis, the CT could become the product of language policy in the legal field and a form of language practice.

Overall, these topics, ranging from the basic concepts of constitutional contents to the potential factors affecting Constitution making, have been the research hotspots in recent years. However, under the guidance of the legal framework of constitutional principles, relatively few studies have taken CT as language practice at the national level and interpreted the characteristics of language policy from a legal perspective.

### CT as a domain of language practice and language rights

Language policy includes the language practices, language beliefs, and management decisions of a community or polity (Spolsky, 2004). In a speech community, language practice means the habitual pattern of selecting among the varieties that make up its linguistic repertoire, while language beliefs (ideology) refer to the beliefs about language and language use; and any specific efforts to modify or influence that practice by any kind of language intervention, planning or management (Spolsky, 2004). The easiest to recognize are policies that exist in the form of clear-cut labeled statements (e.g., a clause) in official documents (e.g., national Constitution), or a language law, or a cabinet document or an administrative regulation (Spolsky, 2004). Therefore, the Constitution has become an important basis for the state authority to make language policies due to its political nature.

A domain is named for a social space, such as home, school, workplace, legal or health institution, or governmental level (city, state, nation) (Spolsky, 2009). The three components of language policy (i.e., language practices, language beliefs, and management decisions of a community or polity) are actually interrelated within a domain (Zhang et al., 2022). Most countries have prescribed language requirements through the Constitution, including the “official language” or the status of other languages, which reflects the general language policy of a nation guaranteed by law. However, there are some countries that so far do not have direct constitutional provisions or language norms. This does not mean that language issues are not important in that country but reflects an invisible language policy. That is, in accordance with national traditions, inaction or other measures, they recognize or allow the official use of only one language, as in the United States, Japan and the United Kingdom. Thus, in the legal domain, language policy in the Constitution is of great concern to most countries around the world.

As mentioned above, the two fundamental and interrelated fields most relevant to language policy in the Constitution are law and linguistics. Constitutions can reflect the shared norms and values of the state (Lerner, 2011), and it is a common practice to use the Constitution as a fundamental solution to the language issues of the state. For example, Azerbaijan promotes the determination of language status through language revival measures (Garibova and Asgarova, 2009), and Ireland has a detailed language plan to assess the implementation of language services (Ó Flatharta, 2015). The language regime in the Constitution is also a means of state language policy (Verschik, 2005; Sokolova et al., 2019). Therefore, the study of language policy in the Constitution has gone from language norms to its application, which is related to the language problems that need to be solved in social development.

Among them, the status of language in LPP is fundamental in language legislation. Language status in the Constitution not only has a symbolic function (Nagy, 2013), but also contributes to the political governance of the country. The French constitutional provision “French is the language of the Republic” is an example of language status planning, which seeks to restore (partially) lost territories through some coercive sphere of society (Bakmand, 1999). Mac Síthigh (2018) elaborated the constitutional implications of the status of language and official language, highlighted the effect of decentralization within the United Kingdom, and made an in-depth study of the relationship between language, territory, and identity. Choudhry (2009) took South Asia as an example, illustrated the possibility of governing linguistic nationalism through constitutional design.

The study of citizens’ basic rights in the Constitution is a fundamental issue in the interdisciplinary study of law and linguistics. Linguistic issues involve specific rights such as the right to freedom which Kadenge and Kufakunesu (2018) has interpreted through the using of indigenous “minority languages” in civil courts, and the right to education, which is one of the basic measures of language legislation with the purpose of building and protecting the state (Saarinen, 2018). Constitutionally speaking, language rights refer to a particular language or small group of languages. Still, it should not be ignored that the main preoccupation addressed by the notion of language rights is the legal situation of speakers of non-dominant languages or where there is no single dominant language (Arzoz, 2007). Therefore, the rights of linguistic minority groups are another subject of language rights that has received increasing attention. For example, minority language governance and regulation (Williams and Walsh, 2019), the fighting for indigenous language rights (Rousseau and Dargent, 2019), the standardization and place-naming planning of sign language (Du Plessis, 2020), and the ideologies of sign languages as well as language policy for revitalization (Lo Bianco, 2020). Taken together, the protection of the language rights of these groups is more specific and targeted, which reflects the real protection of language rights in varying degrees, not just the language provisions in the legislation itself.

### Theory of language policy and planning

Language planning is a body of ideas, laws and regulations (language policy), change rules, beliefs, and practices intended to achieve a planned change (or to stop change from happening) in the language use in one or more communities (Kaplan and Baldauf, 1997). Countries would take political intervention to solve the language problem. One possible method is to write language provisions into the Constitution. Classic language planning is based on the premise that language planning is carried out at the national level, and these plans are formulated for the development of the whole society. The language planning theories of the 1960s and 1970s was formed in a specific political and social context, which left them with unique features. In the 1980s and the following years, many scholars criticized the language planning theory of the previous period. They believed that language planning was beyond the scope of linguistics and should be considered from an interdisciplinary perspective. For example, language planning was actually a political issue in the process of implementation (Nekvapil, 2011). Moreover, Cooper enriched Haugen’s dichotomy, and added acquisition planning on the basis of corpus planning and status planning, by which he made language planning explicitly relevant for applied linguistics. In an era of interdisciplinary integration, based on Cooper’s LPP theory, the research of ecology, sociology and other disciplines were gradually enriched. The theories, frameworks and features of language planning will undoubtedly continue to develop according to the demand for language planning itself in contemporary society. With the development of theories, perspectives and methods of language policy and planning, the trend of interdisciplinary research in this field has become increasingly obvious (Oakes and Peled, 2018; Valle, 2019). Although the Constitution is a political product at the national level, little research has been done on the language policy in the Constitution. Therefore, this paper aims to explore this issue from the interdisciplinary perspective of language policy and law.

In summary, the language policies in the Constitution are not only the focus of research on specific groups or communities, but also the focus of regional studies, which are related to the implementation of the protection of citizens’ basic rights and the smooth progress of national political development. Besides, it is not only a key research issue for language policy, but also a hot spot for jurisprudential norms and state-building concerns. The afore-mentioned studies have enriched our understanding of language issues in CT, but there exist some research gaps at the same time. Firstly, there are more research on the specific language issues of each country and less discussion about the common language problems for the whole world. Most previous studies have focused on case studies of one or a few nations (De Varennes, 1996; Faingold, 2016, 2017; Lagarde, 2019), which might limit the generalizability of research findings, making it difficult to generalize these findings to most countries in the world. Secondly, there exist more studies on the specific language provisions of the Constitution than on the overall analysis of the framework of language policies in Constitution. While some have explored the impact of politics, history, citizenship or language ideology on language legislation in language policy (Blommaert, 1999; Lo Bianco, 2008; May, 2012), it is of great importance to explore and examine other factors in interdisciplinary research, which may enhance our in-depth understanding of language issues and legal framework of language policy. Last but not least, there is a lack of quantitative empirical research using large-scale data, combining perspectives of both law and linguistics. Indeed, the analysis of linguistic Law from the perspective of Comparative Constitutional Law is not a methodological novelty (Ruiz Vieytez, 2004) and some scholars in this field have worked on this approach. More importantly, when drawing more general conclusions regarding the problems and reflections that Linguistic Law must deal with in the future, a global comparative method becomes necessary (Ruiz Vieytez, 2004). Chew et al. (2018) focuses on how different “glob-national” actors have been involved in intended and unintended LPP and their impact on multilingual language use, especially in this globalized world. The global dimension thus offers a new perspective for LPP.

Therefore, to fill these gaps, this study attempts to examine the framework and features of language policies in the Constitution from a global comparative perspective, while focusing on the potential effect of “geographic location” on the relationship between the frequency of language policies and the parts of framework in which they were distributed. Generally, there were three research questions to be addressed in this paper:

RQ1: Is it possible to generalize a framework of language policies based on the CT of all counties in the world?

RQ2: Is there a correlation between the frequency of language policies and the parts of language framework in which they were distributed? In other words, which parts of this framework are closely related to the occurrence of language policies?

RQ3: Is their relationship influenced by the geography of the country (i.e., continent)?

## Methodology

This study was carried out by applying grounded theory (GT), known as Glaser version. GT is a suitable method for qualitative researchers to answer questions like “what is going on in an area?” by generating formal or substantive theory (Corbin and Strauss, 2014). The goal of GT is to discover patterns and understand the social interactions of a group of individuals in the real world (Polit and Beck, 2008). Given that the language provisions in CT are the result of interactions between the different groups of stakeholders and an outcome of their preferences, GT is suitable for understanding this phenomenon. Besides, GT is very helpful when exploring a relatively novel area or trying to obtain a fresh new perspective on a well-known area (Stern, 1994). Nevertheless, to the best of our knowledge, few researchers have applied GT in their constitutional research.

### Data collection

**Figure 1:**
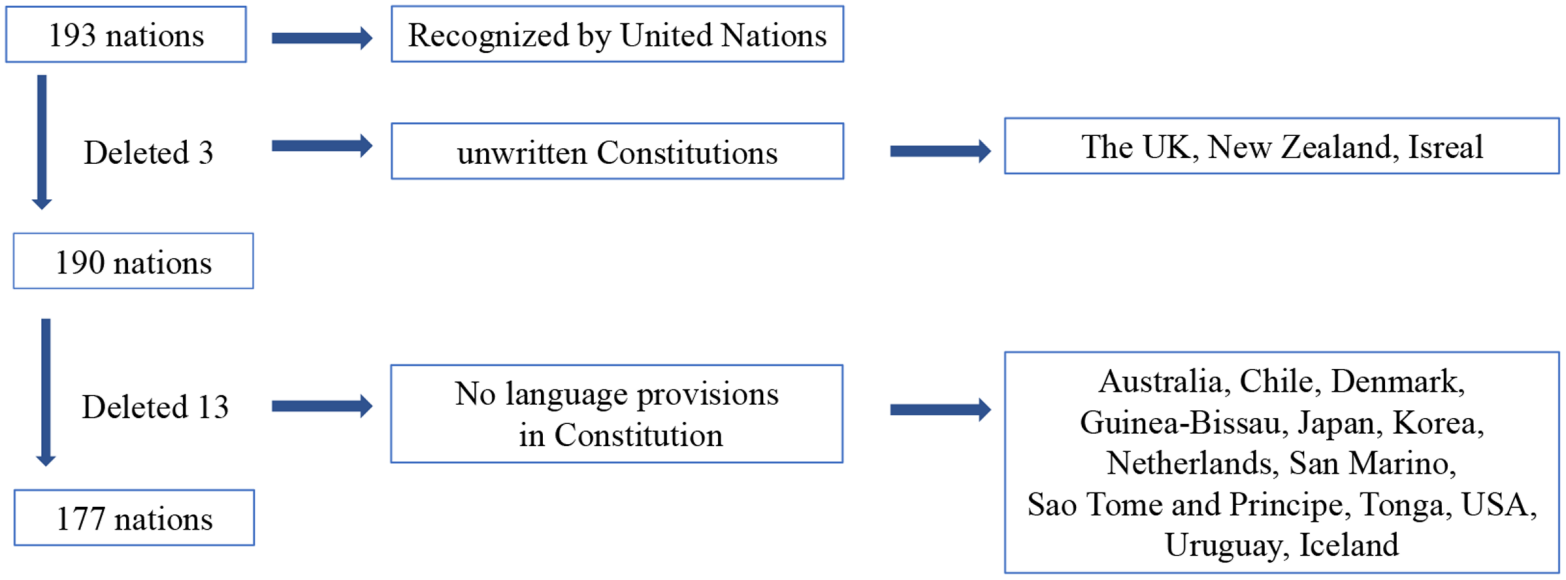
Overview of the screening procedure (*n* = 177 nations).

We searched the constituteproject.org (*Constitute*)^1^ website (January 2022) for the following keywords to capture relevant language provisions: “language(s)” “linguistic(s)” “mother tongue” “multi-lingual” and finally obtained a corpus of CT (*n* = 333,401 words). The use of publicity-available data in this study did not require Institutional Review Board approval. All texts obtained were then classified into six categories, namely, Asia, Africa, Europe, North America, South America and Oceania, according to the classification standards of United Nations, among which 193 countries were recognized by the UN. The final sample data remained 177 countries after several screening rules (see Figure 1).

### Data analysis

The analysis firstly consisted of identifying and describing themes and patterns using the MAXQDA 2020 (VERBI Software, 2019) pro software. Two researchers were involved in the process of data coding. The research team used an iterative and data-driven process of creating codes that were organized into themes representing frequently occurring patterned responses throughout the dataset. Inter-coder consistency was checked for and divergence in code interpretations was eliminated to the best of our ability. After calibrating, interrater reliability was assessed on 177 samples texts (Cohen’s Kappa = 0.915).

Coding is used for analysis of the data collected in the process of grounded theory. During coding, the collected data are analyzed, conceptualized and finally juxtaposed in a new way (Flick, 2009). According to Corbin and Strauss (2014), the coding procedure has three stages: open coding, axial coding and selective coding. These stages are not necessarily separate but complement one another.

During open coding, events, actions and interactions are compared and contrasted and tagged for the purpose of finding similarities and differences. In this stage, data are fractured, analyzed, compared and conceptualized. Conceptualization means that each section of interactions, theories and ideas that are in the related texts get extracted (Corbin and Strauss, 2014). During axial coding, links are established between the concepts and categories that are derived from the open coding stage. The basis of this linking process in axial coding is identification of one core category and classification of other similar codes as its sub-categories (Corbin and Strauss, 2014). Finally, during selective coding, a theory is constructed with a number of abstract codes and there is no need to code new data. At this stage, the codes have become theoretically saturated. These codes are juxtaposed in a logical way based on the coded categories in the first two stages and then the core category is selected. The core category can be selected in two ways: selecting one of the available categories or determining/constructing a new category. Regardless of the method, selecting a core category at this stage requires accurate analysis of the collected data during the first two stages (*ibid.*). MAXQDA software was used in this study to facilitate coding. Upon coding, 1,022 concepts were grouped into 60 subcategories and 7 main categories. The extracted categories were generalized according to principles of constitutional law. Then, the grounded model of the study was developed. The content examples in CT were shown in Table 1. The features of these seven parts were consistent with the constitutional principles. Therefore, these 7 parts were the framework of language policy that we found in CT. The detailed coding process and examples of coding were shown in Supplementary materials 1, 2.

**Table 1:** Examples of language provisions coded into seven parts.

Parts/categories	Sub-categories	CT examples
PR	Safeguarding language status	*Loyalty to Latvia, the Latvian language as the only official language, freedom, equality, solidarity, justice, honesty, work ethic and family are the foundations of a cohesive society*
	Promoting national unity	*The Central African People-Proud of their national unity, linguistic [unity] and of their ethnic, cultural and religious diversity which contribute to the enrichment of their personality*
GP	Regulating language status	*… is/are official language(s)*
	Aiming political principles	*It shall be supported by the work of the experts and assigned the task of providing the necessary requirements to develop the Tamazight language in order to integrate it as an official language in the future*
TS	Regulating language status	*… is/are official language(s)*
	Aiming political principles	*Additionally, the Republic works to protect and promote the national language*
RD	Right to personal liberty	*Any person who is arrested or detained shall be informed at the time of his arrest or detention, in a language that he understands, of the reasons for his arrest or detention*
	Right to language equality	*The freedoms and rights of the individual and citizen can be restricted during states of war or emergency, in accordance with the provisions of the Constitution. The restriction of freedoms and rights cannot discriminate on grounds of sex, race, color of skin, language, religion, national or social origin, property or social status*
SA	Regulation of language use	*The proceedings of Parliament shall be conducted in the English language and such other languages as the National Assembly may prescribe*
	Qualifications for public officials	*A citizen of the Kyrgyz Republic, no younger than 35 years of age and not older than 70 years of age, who has a command of the state language and who has been resident in the republic for no less than 15 years in total may be elected President*
NO	Objectives of culture	*The State shall protect and promote the Khmer language as required*
	Establishment of language institutions	*A Haitian Academy shall be established to standardize the Creole language and enable it to develop scientifically and harmoniously*
SU	Regulatory of texts	*This Constitution will be submitted to referendum. It will be registered and published, in French and in Arabic, in the Official Gazette of the Republic of Djibouti, the text in French will prevail*
	Regulation of language use	*The Indonesian and the English languages shall be working languages within the public administration side by side with official languages as long as it is deemed necessary*

Subsequently, based on the framework of language policies in CT, we further examined the quantitative features of this framework across different parts and countries by using the IBM SPSS 25.0 software. First of all, the descriptive analysis was conducted to summarize the distributions of language policies in each part across nations. Frequencies were calculated to determine the total number of occurrences of language provisions in each part. Afterwards, the Chi-square analysis was conducted to analyze the relationship between the frequency of language provisions and the parts in framework of language policies in CT, as well as the possible influence of geographical location on their relationship.

## Results

### The framework of language policies in CT

There were mainly seven parts constituting the framework of language policies in CT and the distribution of the language provisions in different parts, i.e., seven parts in CT, were presented in Table 2: *Preamble* (2.05%, hereinafter PR), *General Principle* (16.34%, hereinafter GP), *The state* (13.41%, hereinafter TS), *Fundamental Rights and Duties of Citizen* (40.70%, hereinafter RD), *State Authority* (13.21%, hereinafter SA), *National Objectives* (6.65%, hereinafter NO), and *Supplementary* (7.64%, hereinafter SU). The number of language policies in RD was the highest among the seven parts. The descriptive statistics for the distributions of language provisions in each part across nations were shown in Table 3. Specifically, the distribution of language provisions varied across 7 parts, and the RD occupied the largest proportion, which was in line with constitutional principles. Besides, each part contained the core content of language policy, suggesting that language status and language use were still the focus of lawmakers and agencies across countries. The main content and distribution percentages of each part were presented in Table 4.

**Table 2:** Distribution of language provisions across parts.

	PR	GP	TS	RD	SA	NO	SU	Total
Language provisions count	21	167	137	416	135	68	78	1,022
Percentage (%)	2.05	16.34	13.41	40.70	13.21	6.65	7.64	100

**Table 3:** Distribution of language provisions across nations.

	PR	GP	TS	SA	RD	NO	SU
Nations count	19	69	56	88	149	30	43
Percentage (*N* = 177)	10.73	38.98	31.64	49.72	84.18	16.95	24.29

**Table 4:** The main content of each part and its percentage.

Parts	Key words of main content (Top three)	Percentage of each part (%)
PR	Language status	22.7
	National unity	22.7
	Language diversity	18.2
GP	Language status	35.7
	Political principles	20.8
	Official language(s)	14.3
TS	Language status	57.7
	Political principles	13.9
	Official language(s)	10.2
RD	Right to personal liberty	45.5
	Right of language equality	18.4
	Right of culture and language	14.8
SA	Language use	35.7
	Qualifications for public officials	32.2
	Functions of public institution	14.0
NO	Cultural objectives	54.4
	Language management institution	13.2
	Educational objectives	11.8
SU	Regulatory of texts	26.9
	Language use	16.7
	Language status	14.1

### Distribution of language provisions across chapters

**Figure 2:**
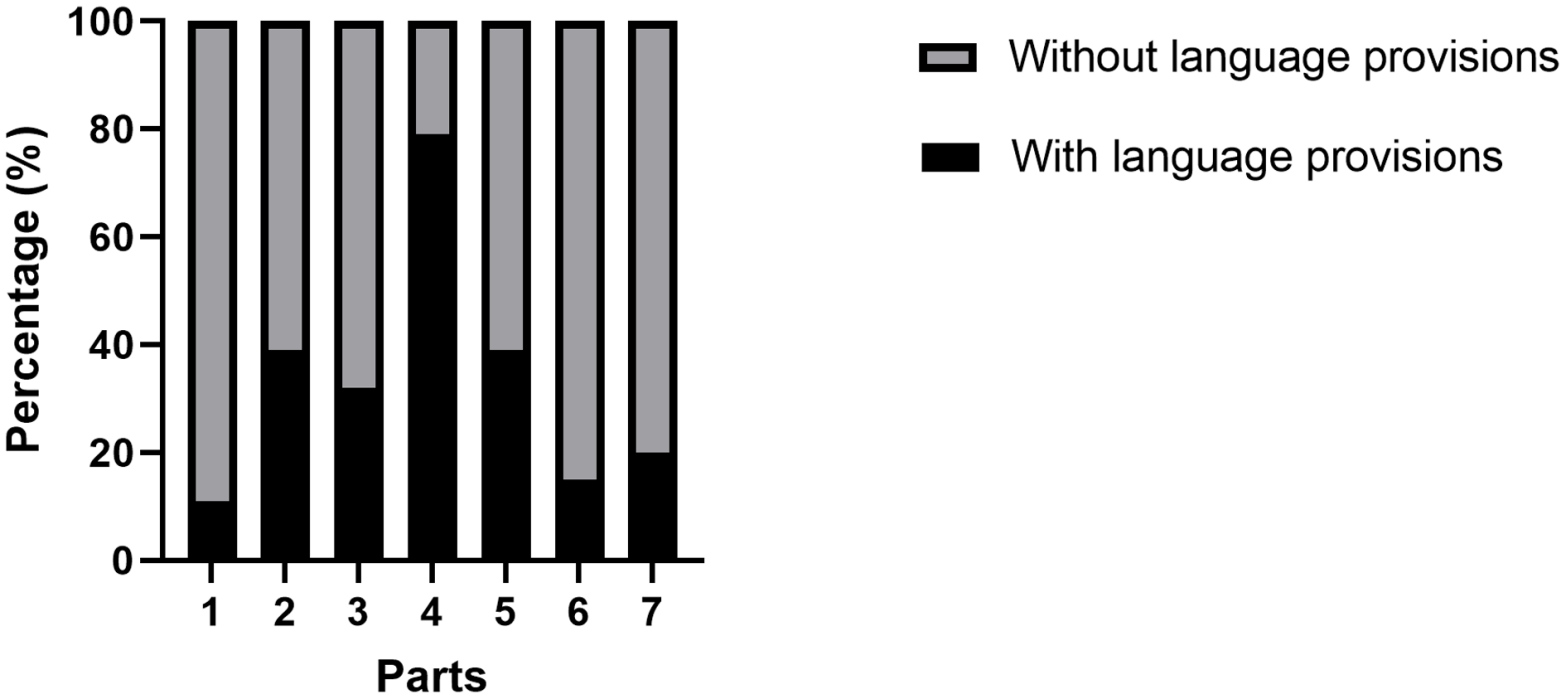
The relationship between constitutional parts and language provisions. “1” = PR; “2” = GP; “3” = TS; “4” = RD; “5” = SA; “6” = NO; “7” = SU.

As shown in Figure 2, the distribution of language provisions varied significantly across parts and RD was the first concern. It was found that there was a significant difference in the frequency of language provisions in different parts of the Constitution (Pearson *χ*^2^ = 253.307, *p* < 0.001). Besides, according to the Cramer’s V coefficient, we found that the correlation between the language provisions and parts was moderately large (*v* = 0.452, *p* < 0.001). Finally, according to the chi-square test (2*C) pairwise comparison, it was found that there was a statistically significant difference in language provisions distribution between the top parts RD and SA (*p* < 0.05). That is, the statistical results conform to the essence of the Constitution.

### Effect of geographical location

In order to further examine whether there were differences in the distribution of language provisions in CT across different regions, we divided countries according to the geographical location (continents) where the Constitution was enacted and analyzed the relationship between language provisions and parts in different regions, respectively.

**Figure 3:**
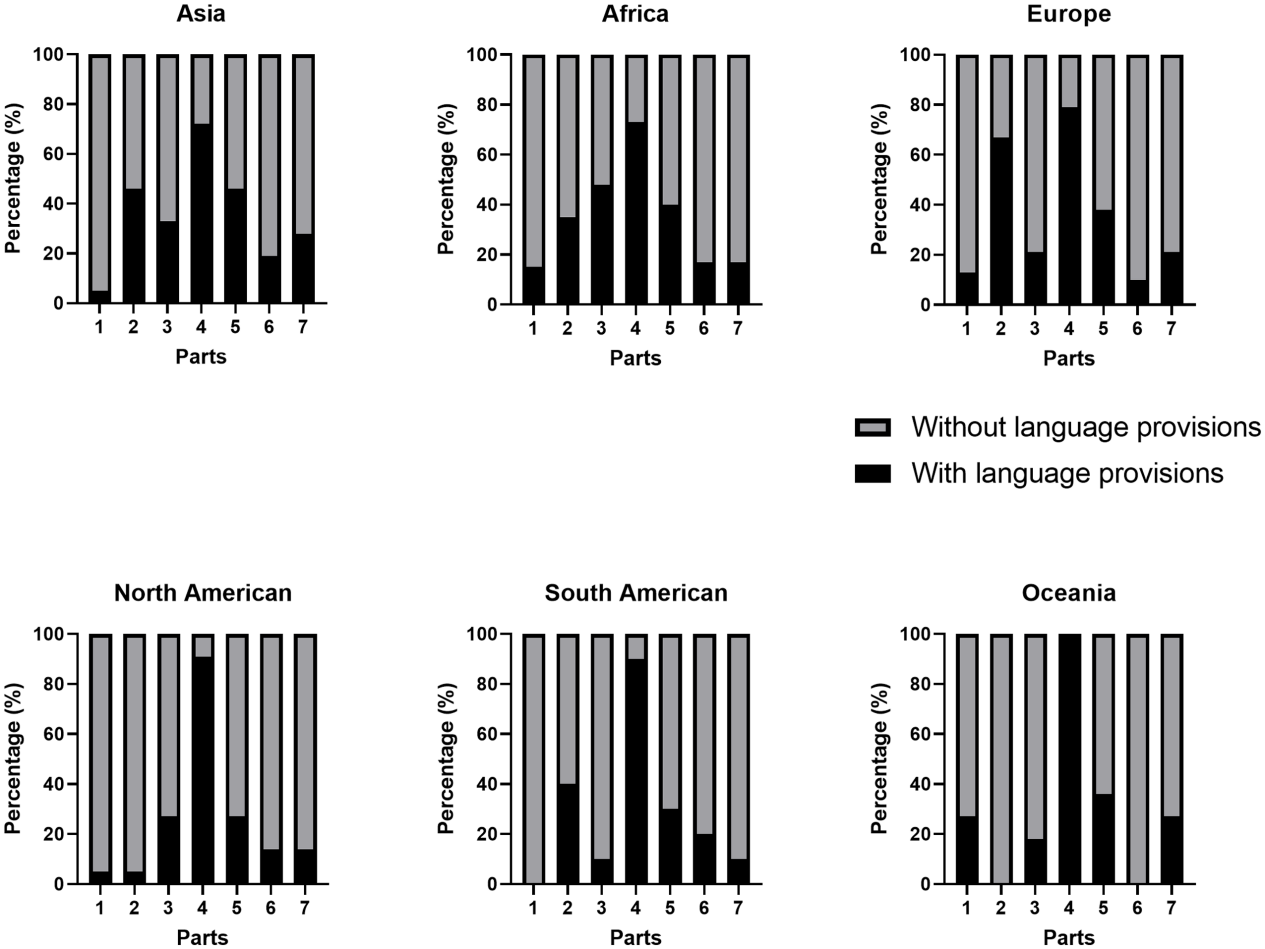
Distribution of language provisions across six continents. “1” = PR; “2” = GP; “3” = TS; “4” = RD; “5” = SA; “6” = NO; “7” = SU.

Overall, we founded that the distribution of language provisions varied significantly across parts in each region (See Figure 3). Firstly, there were significant differences in the frequency of language provisions in different constitutional parts across different continents (Asia: Pearson *χ*^2^ = 54.145, *p* < 0.001, Africa: Pearson *χ*^2^ = 60.683, *p* < 0.001, Europe: Pearson *χ*^2^ = 76.885, *p* < 0.001, North American: Pearson *χ*^2^ = 62.275, *p* < 0.001, South American: Pearson *χ*^2^ = 26.880, *p* < 0.001, and Oceania: Pearson *χ*^2^ = 36.206, *p* < 0.001). Secondly, it was further revealed in Figure 3 that the distribution features of language provisions in each part of the six continents were largely different. In general, it can be inferred that the distribution of language provisions in each part would be affected by geographic location. However, it is worth noting that among the seven parts, RD was the most important part related to language provisions across different continents.

## Discussion and implications

We conducted a comparative study of language policies in different CT, based on 177 countries, using qualitative and quantitative methods. We founded that (1) there were seven parts of the CT dealing with language policies; (2) there existed significant differences in the frequency of language policies in seven parts of the CT, and that (3) the geographical location where the Constitution was enacted affected the distribution of language policies across parts.

Previously, there was an established framework of CT (Maarseveen, 2007) from the perspective of law, consisting of political structure, legal system, constitutional form, values and norms, cultural systems, and nation-building. However, the framework of language policies in CT should also be established since most countries in the world have written language policies into their Constitutions. In response, this study has found a framework of language policies in CT based on the principles and values of the global Constitution. In terms of LPP theoretical development, the results of this study have validated Lo Bianco’s (2008) concept of language policy “public text policy” as a social behavior. Besides, it has enriched the content of Fitzsimmons-Doolan’s (2019) research and provided a novel perspective for researchers to further understand the characteristics of language policy in the special register of LPP. By finding a framework of language policies in the Constitution, this study displayed a full interdisciplinary picture for researchers in the field of linguistics and law who are interested in language legislation.

Comparative constitutional study focused on the essential relationship between RD and SA. The statistical analysis results of this research showed that these two parts of this paper were relevant. Therefore, to a certain degree, our findings are consistent with the principles and characteristics of the Constitution. As for second research question, we aimed to reveal the correlation between the frequency of language policies and the parts of language framework in which they were distributed. Firstly, we found that the number of language provisions in the RD was the largest among seven parts. As shown in Figures 2, 3, it was not only the most prominent part of the framework, but also the largest number of language provisions in each continent. This was a new feature of language policy in CT. In the early days of the establishment of a nation state, language status was usually determined in the Constitution, highlighting the symbolism of language policy, because it reflected the country’s primary political goal. But our findings showed that the language policy in the CT was more functional to protect citizens’ rights and duties. Another new finding was that the provisions on language status were usually distributed in two parts, GP and TS. It was generally believed that the provisions on language status were very important, but few studies have explored the part in which such provisions were written. Our research found that although the themes of GP and TS were almost the same (see Table 4), we did not combine them into a whole, because we found that each part of CT has its own characteristics, intrinsic values and norms based on the nature of the Constitution. Specifically, TS placed more emphasis on the national sovereignty and status symbol, while GP was more regarded as general rules. In addition, this behavior is also related to the constitutional characteristics of countries in different regions. Just as Colón-Ríos (2011) prescribed the characteristics of Latin American Constitutions, “It is in the third and fourth waves that the multi-cultural and multi-lingual character of the region was able to make its way into constitutional law. During these waves, constitution-makers decided to alter the ways in which their countries described themselves to the world, moving beyond the idea of a single national culture, and gave constitutional recognition to cultural difference.” We, therefore, suggested that GP and TS were two parts with different functions. From a legal perspective, the GP and the TS were special parts in language policy framework, but according to linguists, they embodied the symbolism of the language policy in the Constitution. Just as Cooper (1989) said “In many cases, the function of a language is specified constitutionally … But it may be useful to distinguish two other types of official language: a language which a government uses as a medium for its day-to-day activities and a language which a government uses as a medium for symbolic purposes, i.e., as a symbol of the state.” Furthermore, official language has more than an instrumental and symbolic role in the Constitution. It has been argued that the designation of one or more languages as official did not necessarily or automatically entail significant legal consequences (Turi, 2012). The legal significance of making a language as an official language depends on the effective legal treatment accorded to the language. When the state determines the status of a language, it is usually also guided by state policy, where the state guarantees the status and use of the language, in which case the language takes on a social function, combining with economic, political, and cultural factors and performing a dynamic role. In the domain of legal contexts, there were systematic differences in the functionality of language policies in the legal register, suggesting that language functions in language policy legal texts differed from previous studies, which only treated language provisions as symbols. Language use and language status were the basis and focus of language legislation, to highlight their functionality. In particular, the cultural and social functions of language provisions were also playing a role. More and more countries have begun to take effective measures to promote and maintain language in order to strengthen nation-building and solidarity. The measures they usually take could be reflected in the language policies in RD, which has the largest proportion of language provisions in the CT, highlighting the core principle of the Constitution, that is, the basic rights of citizens guaranteed by the state. Protecting citizens’ language rights is the starting point for the state authority to exercise language power, and it can also reflect its specific measures to protect citizens’ language rights. These measures reflect the functionality of language policies.

Finally, the language policies of CT have obvious characteristics of localization and contextualization. Previous study rarely took location as an important influential factor, but there existed large differences in the distribution of language policies across different continents (Figure 3). Therefore, we cannot conclude that the framework of language policies and its features in the constitutional context are fixed. In fact, formulating constitutional language policies for each nation would be a political objective. Just as Blommaert and Verschueren (2022) mentioned that the role of language in nationalist ideology was, to a large extent, political. In other words, language has both normative and political features. If we look at the development history of the world Constitution and the content of their provisions, we will find that all Constitutions, regardless of their substance or form, are related to democracy. The Constitutions actually express the aspirations, principles and means of the ruling class for democratic management of the state and all aspects of social life and reflects the purpose to be achieved by implementing these aspirations, principles and means. The language policies in GP, TS, RD, and SA parts are precisely aimed at the will and interests of the ruling class to establish a democratic country. The language policies in the Constitution also mean language choice to a certain extent and reflect the determination of the status of language, the scope of language use, citizen’s choice of language for expression, education and communication, and the guarantee of the fundamental rights of the common people by the state institutions through the choice of language through status planning. The Constitution is the embodiment of national consciousness, national political language policy and national attitude toward language. Linguistic awareness, which plays an important role in the Constitution, reflects the political compromise and consensus on the development of the state. Therefore, the Constitution is a consensual and legitimate commitment, and linguistic awareness has become one of the factors affecting the constitutional order.

Overall, the findings of this study indicate that interdisciplinary research could expand the scope of research. Theoretically, we combined Cooper’s language policy theory with legal principles, enriching the characteristics of language policy. By comparing the global CT, we have discovered the framework and features of language policies in the Constitution. On this basis, we can help determine the current situation and development trend of global language issues in the Constitution. Moreover, the legal policymaking is a multi-party negotiation process, and the literal meaning of CT encompasses not only the regulation of language, but also the language ideology of stakeholders. The findings of this study could also be used to predict the trend of LPP in the legal domain.

## Conclusion

The main goal of this study was to uncover the framework of language policies in CT through a hybrid qualitative and quantitative approach. It turned out that all continents in the world regulated language provisions differently and granted language rights or language status according to their own needs and language surroundings. Regulations of language provisions in RD was an essential part of the Constitution and played an indispensable role in the constitutional framework.

The main theoretical contribution of this paper is to enrich Cooper’s LPP classification from an interdisciplinary perspective and endow it with new connotations. Status planning refers to which discipline occupied the main position, corpus planning refers to which content was the core, and acquisition planning refers to what measures the country took to systematically work across disciplines (Oakes and Peled, 2018). The new findings suggest that legal principles are the most important, which represents the status planning. The seven parts in the framework are the core content of CT, representing the corpus planning. Additionally, policy-makers need to strengthen the language awareness based on the geographical location of each country, and to formulate language policies in the Constitution in line with its own national conditions, which reflects the acquisition planning.

Meanwhile, this study has some practical implications for the primary stakeholders of language management in each country. Firstly, the framework of language policies in the Constitution could facilitate policymakers at the national level to formulate or adjust language policies in the Constitution or other language-related laws. Secondly, this study has linked language power with value, indicating that some nations may be able to improve their political capacity by managing their language policies in CT. Finally, in the age of globalization, the authorities need not only to stabilize their domestic governance, but also to keep pace with the world’s constitutional legitimacy.

However, it is worth noting that there are several limitations in this study. First, due to technical retrieval problems, we could only find the latest valid samples, but cannot trace the previous ones. Therefore, we hope that more diachronic comparative studies could be conducted in the future. Second, this research has explored the distribution features of language policies from the perspective of geographical location, but there may be other factors, such as national politics, doctrine, etc., worthy of further discussion. In addition, from the perspective of language policy, there are many factors that could affect the process of formulation of language policies, such as international treaties, language ideology, language management, and even language tradition and value. These are also factors that cannot be ignored in the discussion of constitutional language policy framework. Last but not least, we have selected the English corpus from official databases in order to ensure the authority and validity of the data. However, at the same time, we may ignore the specificity of certain countries whose official language is not English. Specifically, the interpretation of the words or texts translated into English may have a different meaning in the language of the original version. Indeed, this is a problem that is currently insurmountable when conducting global comparative studies of the Constitutions, and we hope to overcome this problem through technological innovation in the future.

## Data availability statement

The raw data supporting the conclusions of this article will be made available by the authors, without undue reservation.

## Author contributions

YH conceived the project and edited the manuscript. CZ conducted the study, analyzed the data, and drafted the original manuscript with the help of YH and RZ. All authors reviewed the manuscript and approved the final version of the manuscript for submission.

## Funding

This research was supported by the Ministry of Education of China’s major research project on philosophy and social sciences, “Construction and Comparative Research on the Comprehensive Resource Bank of World Language Policy” (Project No. 15JZD047), and “Disciplinary Innovation and Talent Introducing Program” (111 Project B20081) of Higher Education Institutions.

## Conflict of interest

The authors declare that the research was conducted in the absence of any commercial or financial relationships that could be construed as a potential conflict of interest.

## Publisher’s note

All claims expressed in this article are solely those of the authors and do not necessarily represent those of their affiliated organizations, or those of the publisher, the editors and the reviewers. Any product that may be evaluated in this article, or claim that may be made by its manufacturer, is not guaranteed or endorsed by the publisher.

## Footnotes

^1^The website, which offers the possibility to read and/or download the whole text of each Constitution of almost nations in the world, maintained by the University of Texas at Austin’s Comparative Constitutions Project. In addition, it offers the possibility of conducting queries on a number of issues, such as provisions regarding the official language, and the first time they mentioned. Constitute includes the currently-in-force Constitution for nearly every independent state in the world, as well as some draft and historical texts.
