# Supplementary figures and images for: The framework and features of language policies in global constitutional texts
Source: Front Psychol. 2023 Jan 4;13:1064034. doi: 10.3389/fpsyg.2022.1064034 (PMC9846269; doi:10.3389/fpsyg.2022.1064034)

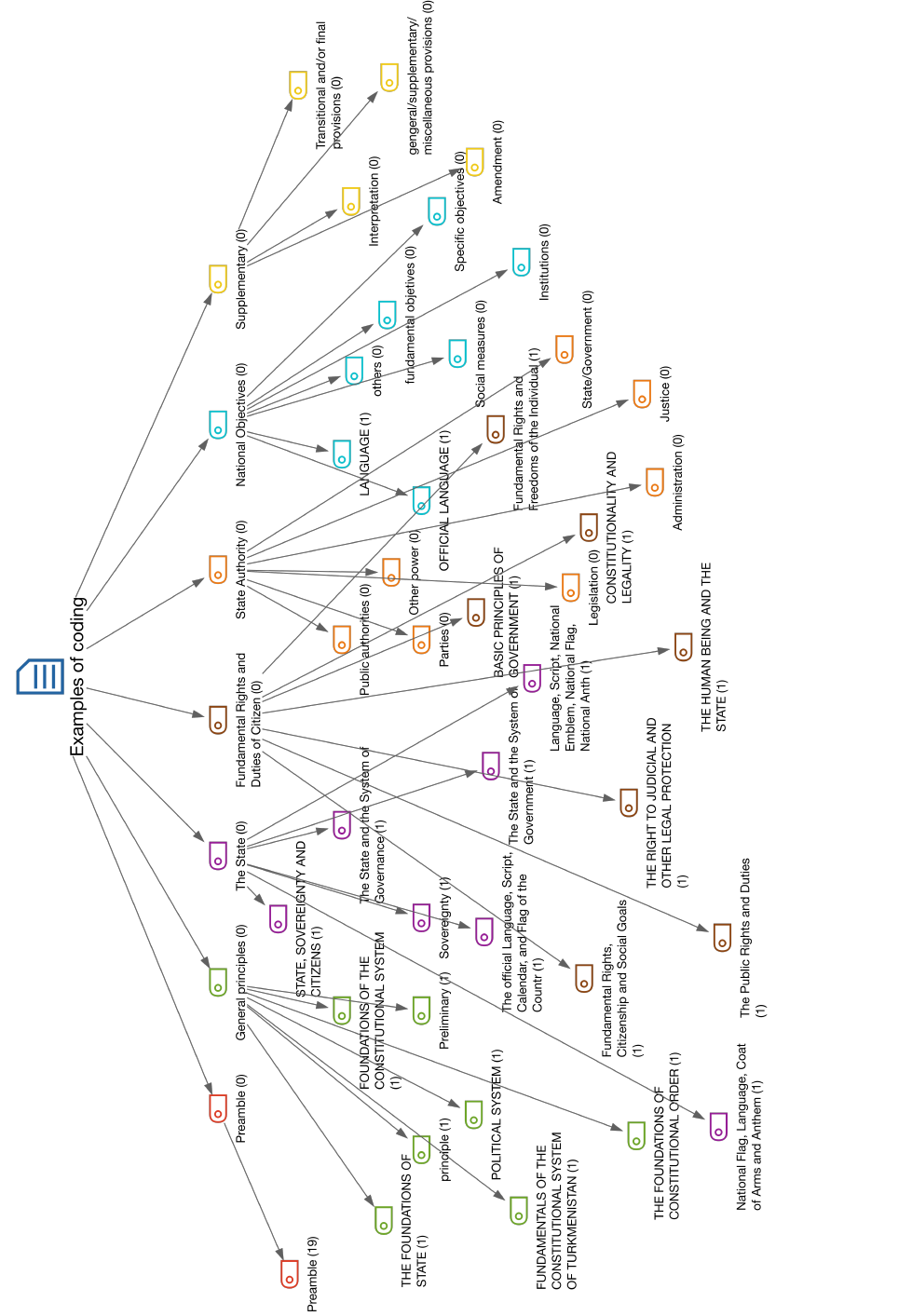

Supplement: Supplementary file 1 [file Image_1.TIF]
